# Neutralization sensitivity, fusogenicity, and infectivity of Omicron subvariants

**DOI:** 10.1186/s13073-022-01151-6

**Published:** 2022-12-29

**Authors:** Xue-Jun Wang, Lin Yao, Hong-Yun Zhang, Ka-Li Zhu, Jing Zhao, Bing-Dong Zhan, Yi-Ke Li, Xue-Juan He, Cong Huang, Zhuang-Ye Wang, Ming-Dong Jiang, Peng Yang, Yang Yang, Guo-Lin Wang, Sheng-Qi Wang, Er-Hei Dai, Hui-Xia Gao, Mai-Juan Ma

**Affiliations:** 1 State Key Laboratory of Pathogen and Biosecurity, Beijing Institute of Microbiology and Epidemiology, Beijing, China; 2 Department of Respiratory and Critical Care, The Second Medical Center & National Clinical Research Center for Geriatric Diseases, Chinese PLA General Hospital, Beijing, China; 3 Department of Epidemiology and Biostatistics, School of Public Health, Anhui Medical University, Hefei, China; 4 Quzhou Center for Disease Control and Prevention, Quzhou, China; 5 School of Public Health, Zhengzhou University, Zhengzhou, China; 6 Dezhou Center for Disease Control and Prevention, Dezhou, China; 7 The Fifth Hospital of Shijiazhuang, Hebei Medical University, Shijiazhuang, China

**Keywords:** SARS-CoV-2, Omicron subvariants, Breakthrough infection, Vaccination neutralization, Fusogenicity, Infectivity

## Abstract

**Background:**

The emergence of SARS-CoV-2 Omicron subvariants has raised questions regarding resistance to immunity by natural infection or immunization. We examined the sensitivity of Delta and Omicron subvariants (BA.1, BA.1.1, BA.2, BA.2.12.1, BA.4/5, and BA.3) to neutralizing antibodies from BBIBP-CorV-vaccinated and BBIBP-CorV- or ZF2001-boosted individuals, as well as individuals with Delta and BA.1 breakthrough infections, and determined their fusogenicity and infectivity.

**Methods:**

In this cross-sectional study, serum samples from two doses of BBIBP-CorV-vaccinated individuals 1 (*n* = 36), 3 (*n* = 36), and 7 (*n* = 37) months after the second dose; BBIBP-CorV- (*n* = 25) or ZF2001-boosted (*n* = 30) individuals; and fully vaccinated individuals with Delta (*n* = 30) or BA.1 (*n* = 26) infection were collected. The serum-neutralizing reactivity and potency of bebtelovimab were assessed against D614G, Delta, and Omicron subvariants (BA.1, BA.1.1, BA.2, BA.2.12.1, BA.4/5, and BA.3) through a pseudovirus neutralization assay. The fusogenicity and infectivity of D614G, Delta, and Omicron subvariants were determined by cell-cell fusion assay and pseudovirus infection assay, respectively.

**Results:**

Omicron subvariants markedly escaped vaccine-elicited neutralizing antibodies after two doses of BBIBP-CorV with comparable efficiency. A third dose vaccination of BBIBP-CorV or ZF2001 increased neutralizing antibody titers and breadth against Delta and three Omicron subvariants. Delta and BA.1 breakthrough infections induced comparable neutralizing antibody titers against D614G and Delta variants, whereas BA.1 breakthrough infections elicited a stronger and broader antibody response against three Omicron subvariants than Delta breakthrough infections. BA.2.12.1 and BA.4/5 are more resistant to immunity induced by breakthrough infections. Bebtelovimab had no significant loss of potency against the Delta and Omicron subvariants. Cell culture experiments showed Omicron subvariants to be less fusogenic and have higher infectivity than D614G and Delta with comparable efficiency.

**Conclusions:**

These findings have important public health implications and highlight the importance of repeated exposure to SARS-CoV-2 antigens to broaden the neutralizing antibody response against Omicron subvariants.

**Supplementary Information:**

The online version contains supplementary material available at 10.1186/s13073-022-01151-6.

## Background

As of the end of 2021, five severe acute respiratory syndrome coronavirus 2 (SARS-CoV-2) variants of concern (VOCs) have emerged, including Alpha, Beta, Gamma, Delta, and Omicron [1]. Among these five VOCs, the SARS-CoV-2 Omicron variant (B.1.1.529 or BA.1) is highly divergent from the prototype virus (Wuhan-Hu-1). Omicron comprises several subvariants, including BA.1, BA.2, BA.3, BA.4, and BA.5 [2, 3]. The original subvariant BA.1 and its derivative BA.1.1 with an extra spike (S) R346K substitution rapidly became dominant globally after its first detection in South Africa in November 2021 [4]. After a surge of BA.1, subvariant BA.2 outcompeted BA.1 and has become the most dominant variant worldwide. Thereafter, BA.2 subvariants that harbor the substitution at the L452 residue of the S protein, such as BA.4 and BA.5 (BA.4/5, share an identical spike) that emerged from South Africa and BA.2.12.1 that emerged in the USA, were frequently detected [3, 5]. The new subvariants BA.2.12.1 and BA.4/5 show more infectious potential than BA.2 [6]. As of the beginning of September 2022, Omicron BA.5 is the most predominant SARS-CoV-2 variant in the world. These Omicron subvariants share multiple mutations, but each also has unique mutations [2], which are expected to be associated with different antigenic properties.

Neutralizing antibodies are strongly predictive of the degree of immune protection against symptomatic SARS-CoV-2 infection [7]. Inactivated vaccines, such as BBIBP-CorV by Sinopharm, and protein subunit vaccines, such as ZF2001 (a tandem-repeat of the dimeric receptor-binding domain [RBD] of the SARS-CoV-2 Wuhan-Hu-1 S protein-based protein subunit vaccine by Anhui Zhifei Longcom, China), have been widely used in China and several other countries [8], and boosting is available in China. Previous studies have reported that Omicron subvariants BA.1, BA.2, and BA.3 showed substantial resistance to neutralizing antibodies induced by vaccination, natural infection, and therapeutic monoclonal antibodies [9–22]. However, the susceptibility of the Omicron subvariants BA.2.12.1, BA.4, and BA.5 to neutralizing antibodies elicited by prime and booster vaccination with BBIBP-CorV and ZF2001 and breakthrough infections and their fusogenicity and infectivity remain largely unexplored. Here, we measured pseudovirus-neutralizing antibodies against D614G, Delta, and Omicron subvariants (BA.1, BA.1.1, BA.2, BA.2.12.1, BA.4/5, and BA.3) in fully vaccinated individuals with two doses of BBIBP-CorV, individuals with a third-dose booster vaccination of BBIBP-CorV or ZF2001, and fully vaccinated individuals with Delta or BA.1 breakthrough infection. In addition, we investigated the fusogenicity and infectivity of Omicron subvariants in vitro compared with the SARS-CoV-2 ancestral D614G and Delta variants.

## Methods

### Human subjects

We conducted a cross-sectional study to investigate the effect of vaccination and breakthrough infection on the cross-variant neutralization capacity of human sera after vaccination or infection. We enrolled SARS-CoV-2 naïve individuals with two or three doses of BBIBP-CorV and two doses of BBIBP-CorV boosted by ZF2001, as well as previously vaccinated individuals with Delta or BA.1 breakthrough infection. Vaccine sera of 36 (22 females/14 males with a median age of 44.5 years; interquartile range [IQR], 36.3–49.8), 36 (19 females/17 males with a median age of 42.0 years; IQR, 31.5–52.8), and 31 (20 females/11 males with a median age of 45.0 years; IQR, 39.0–50.0) individuals who had two doses of BBIBP-CorV were collected a median of 20.5 (one month), 91.0 (three months), and 221.0 (seven months) days after the second dose, respectively. Vaccine sera of 25 (12 females/13 males with a median age of 46.0 years; IQR, 38.5–52.0) and 30 (19 females/11 males with a median age of 39.0 years; IQR, 34.3–47.3) individuals who were boosted with the third dose of BBIBP-CorV or ZF2001 were collected a median of 21.0 and 28.0 days after the third dose vaccination, respectively.

Sera of 30 individuals (15 females/15 males with a median age of 39.0 years; IQR, 34.0–48.8) with Delta breakthrough infections were collected when they were discharged from the Fifth Hospital of Shijiazhuang, Hebei Medical University (Shijiazhuang, China), with a median of 41.0 days post-symptom onset or PCR positive result. Sera of 26 individuals (7 females/19 males with a median age of 32.5 years; IQR, 16.0–36.8) with BA.1 breakthrough infections were collected when they were discharged from the hospital, with a median of 15.5 days post-symptom onset or PCR positive result. Sera were separated by centrifugation at 2000 rpm for 10 min, aliquoted into three cryovials, and cryopreserved at − 80 °C until use. Sera were heat inactivated at 56 °C for 60 min prior to use in neutralization assays. Serum-neutralizing capability was characterized using pseudovirus neutralization assays. The details of the demographic information (e.g., age and sex) and sample collection time points of different cohorts are summarized in Additional file 1: Tables S1 and S2.

Breakthrough infection was defined as fully vaccinated individuals being diagnosed with SARS-CoV-2 infection [23, 24]. Full vaccination was defined as when the second shot of the BBIBP-CorV vaccination was administered at least 14 days before symptom onset or a positive PCR test for SARS-CoV-2 [23, 24]. All individuals with breakthrough infection had sequence-confirmed Delta and BA.1 infection or PCR-confirmed symptomatic disease occurring while in isolation and direct contact with Delta or BA.1 sequence-confirmed cases.

### Cell lines

Human embryonic kidney 293T (HEK-293T, CRL-3216, ATCC) cells were cultured at 37 °C and 5% CO_2_ in Dulbecco’s modified Eagle’s medium (DMEM, Gibco) containing 10% (v/v) heat-inactivated fetal bovine serum (FBS, Gibco) and supplemented with 1% penicillin–streptomycin (Gibco). Cells were disrupted at the confluence with 0.25% trypsin in 1 mM EDTA (Solarbio) every 48–72 h. HEK-293T cells over-expressing human angiotensin-converting enzyme 2 (293T-ACE2) and HeLa-ACE2 cells kindly provided by Dr. Lin-Qi Zhang at Tsinghua University were cultured under the same conditions.

### Monoclonal antibody

Bebtelovimab (LY-CoV1404) was purchased from AtaGenix (Wuhan, China).

### Spike plasmid pseudovirus production

Codon-optimized cDNA encoding the SARS-CoV-2 S glycoprotein of D614G, Delta, BA.1, BA.1.1, BA.2, BA.2.12.1, BA.4/5, or BA.3 was synthesized by GenScript and cloned into pCDNA3.1. Compared to the Wuhan-Hu-1 strain S protein amino acid sequence, the following changes were included in the respective expression plasmids: D614G (D614G), Delta (T19R, G142D, Δ156–157, R158G, L452R, T478K, D614G, P681R and D950N), BA.1 (A67V, Δ69–70, T95I, G142D, Δ143-145, Δ211, L212I, 214EPE, G339D, S371L, S373P, S375F, K417N, N440K, G446S, S477N, T478K, E484A, Q493R, G496S, Q498R, N501Y, Y505H, T547K, D614G, H655Y, N679K, P681H, N764K, D796Y, N856K, Q954H, N969K, L981F), BA.1.1 (A67V, Δ69–70, T95I, G142D, Δ143-145, Δ211, L212I, 214EPE, G339D, R346K, S371L, S373P, S375F, K417N, N440K, G446S, S477N, T478K, E484A, Q493R, G496S, Q498R, N501Y, Y505H, T547K, D614G, H655Y, N679K, P681H, N764K, D796Y, N856K, Q954H, N969K, L981F), BA.2 (T19I, Δ24–26, A27S, G142D, V213G, G339D, S371F, S373P, S375F, T376A, D405N, R408S, K417N, N440K, S477N, T478K, E484A, Q493R, Q498R, N501Y, Y505H, D614G, H655Y, N679K, P681H, N764K, D796Y, Q954H, N969K), BA.2.12.1 (BA.2+L452Q+S704L), BA.4/5 (T19I, Δ24–26, A27S, Δ69–70, G142D, V213G, G339D, S371F, S373P, S375F, T376A, D405N, R408S, K417N, N440K, L452R, S477N, T478K, E484A, F486V, Q498R, N501Y, Y505H, D614G, H655Y, N679K, P681H, N764K, D796Y, Q954H, N969K), and BA.3 (A67V, Δ69–70, T95I, G142D, Δ143–145, Δ211, L212I, G339D, S371F, S373P, S375F, D405N, K417N, N440K, G446S, S477N, T478K, E484A, Q493R, Q498R, N501Y, Y505H, D614G, H655Y, N679K, P681H, N764K, D796Y, Q954H, N969K). All plasmid spike sequences were verified by Sanger sequencing.

Pseudovirus particles were generated by co-transfecting HEK-293T cells (ATCC) with human immunodeficiency virus backbones expressing firefly luciferase (pNL4-3-R-E-luciferase) and the pcDNA3.1 vector encoding either D614G or mutated S protein (Delta and Omicron subvariants) plasmids. The medium was replaced with fresh medium at 24 h, and the supernatants were harvested at 48 h post-transfection and clarified by centrifugation at 300×*g* for 10 min before being aliquoted and stored at − 80 °C until use.

### Pseudovirus neutralization assay

A SARS-CoV-2 pseudovirus neutralization assay (pVNT) was performed as described [25], with the target cell line HeLa over-expressing hACE2 orthologs. All viruses were first titrated to normalize the viral input between assays. Duplicate 3-fold 8-point serial dilutions of heat-inactivated sera (starting at 1:30) or LY-CoV1404 (1.0 μg ml^−1^) were incubated with 500–1000 TCID_50_ of SARS-CoV-2 pseudotyped virus for 1 h at 37 °C and 5% CO_2_. Subsequently, 1 × 10^4^ HeLa-ACE2 cells per well were added and incubated at 37 °C and 5% CO_2_ for 48 h. Afterward, the supernatant was removed, and the cells were lysed using passive lysis buffer (Vazyme) for 3 min at room temperature. The lysates were transferred to an opaque white 96-well plate, and reconstituted luciferase assay buffer (Vazyme) was added and mixed with each lysate. Luminescence was measured immediately after mixing using a GloMax 96 Microplate Luminometer (Promega). The neutralization titer (NT_50_) was determined by luciferase activity with a four-parameter non-linear regression inhibitor curve in GraphPad Prism 8.4.2 (GraphPad Software). NT_50_ was defined as the highest reciprocal serum dilution causing a 50% reduction in relative light units. A sample with NT_50_ values no more than 30 (the detectable limit) was considered negative for neutralizing antibodies and was assigned a nominal value of 10 in geometric mean titer (GMT) calculations, which is the lowest serum dilution factor used in the pseudovirus neutralization assay.

### Cell–cell fusion assay

The cell-fusion activity of SARS-CoV-2 S variants was evaluated with a cell–cell fusion reporting assay based on split mNeonGreen and NanoLuc proteins. Briefly, HEK-293T cells at 80% confluence were co-transfected with pNGJS (NG-bJun-SmBiT expression) and pLVX-hACE2-IRES-tdTomato plasmids at a 1:1 ratio for 24 h at 37 °C and 5% CO_2_, for preparing target cells. HEK-293T cells co-transfected with pCGFL (CG-bFos-LgBiT expression) and S plasmids at a 1:1 ratio for 24 h at 37 °C and 5% CO_2_ to prepare effector cells, and a no-S expression plasmid was co-transfected as the negative control. Following 24 h of transfection, the cells were detached and resuspended in fresh DMEM containing 5% FBS. Subsequently, 1.0 × 10^4^ target cells per well were added to a 96-well plate, and 1.0 × 10^4^ effector cells per well were added to the 96-well plate with target cells. The mNeonGreen and tdTomato fluorescent proteins were confirmed by an IX71 fluorescence microscope (Olympus), and the luciferase activity was measured 6 h after co-culturing using the Nano-Light Luciferase Assay Kit (Meilunbio, Dalian, China).

### Western blot analysis

Western blotting was performed as previously described. HEK-293T cells co-transfected with the S variant plasmids and pCGFL were used. Briefly, cells were lysed in RIPA buffer containing protease inhibitors (Solarbio, Beijing, China). The protein concentration was measured using a BCA Protein Assay Kit (Beyotime, Shanghai, China). Polyacrylamide gel electrophoresis and protein transfer to nitrocellulose membranes (EMD Millipore, Billerica, MA, USA) were carried out following standard protocols. Rabbit anti-SARS-CoV-2 S IgG antibody (Sino Biological, Beijing, China) or mouse anti-β-actin monoclonal antibody (Solarbio, Beijing, China) was used for immunodetection according to the manufacturer’s instructions. Protein bands were visualized using a Novex™ ECL substrate reagent kit (Thermo Fisher Scientific, Inc.) with the ECL imaging system (Tanon, Shanghai, China).

### Pseudovirus infection assay

For analysis of pseudovirus infection ability, 293T-hACE2 cells were added to 96-well plates and infected with the indicated pseudoviruses collected between 36 and 48 h post-transfection. The medium was replaced with fresh medium at 12 h post-infection. Luciferase activity was detected at 60 h post-infection with a Steady-Lumi™ II Firefly Luciferase Assay Kit (Beyotime, Shanghai, China). The pseudovirus infection ability was calculated based on the relative luciferase activity and normalized to the pseudovirus RNA genome copy numbers determined by real-time reverse transcription-polymerase chain reaction (RT–PCR).

### Quantitation of pseudovirus by RT–PCR

To determine the genome copy numbers of pseudoviruses, lentiviral RNA was extracted with the TaKaRa MiniBEST Viral RNA/DNA Extraction Kit Ver.5.0 (TaKaRa, Beijing, China). After DNase I digestion, the relative genome RNA copy numbers of pseudoviruses were analyzed with RT–PCR using the One-Step TB Green PrimeScript PLUS RT–PCR kit (Takara, Beijing, China) according to the manufacturer’s instructions. The primers were as follows: LV-F 5′-TAGTAGGAGGCTTGGTAGGT-3′ and LV-R 5′-GTGGGTCTGAAACGATAATGG-3′. The following cycling conditions were used: 42 °C for 5 min, 95 °C for 10 s followed by 40 cycles of 95 °C for 5 s and 60 °C for 34 s, 95 °C for 15 s and 60°C for 1 min, and 95°C for 15 s.

### Statistical analysis

Data and statistical analyses were performed using GraphPad Prism 8.4.2 (La Jolla, CA, USA) and R v4.0.5. Fold changes in serum-neutralizing activity were measured by comparing GMT. Fold changes in monoclonal antibodies were determined by comparing individual IC_50_ or IC_90_ values and then averaging the individual fold changes for reporting. Categorical and continuous variables in supplementary tables were analyzed using the chi-square test, Fisher’s exact test, or Kruskal–Wallis test. The Friedman test with the false discovery rate method was used for multiple comparisons for paired groups, while the Kruskal–Wallis test with the false discovery rate method was used for multiple comparisons for unpaired groups. The Wilcoxon rank-sum test was used for unpaired comparisons between the two groups. All statistical tests were 2-sided with a significance level of 0.05.

## Results

### Two doses of the BBIBP-CorV vaccine induced limited neutralization activity against Delta and Omicron subvariants

**Fig. 1:**
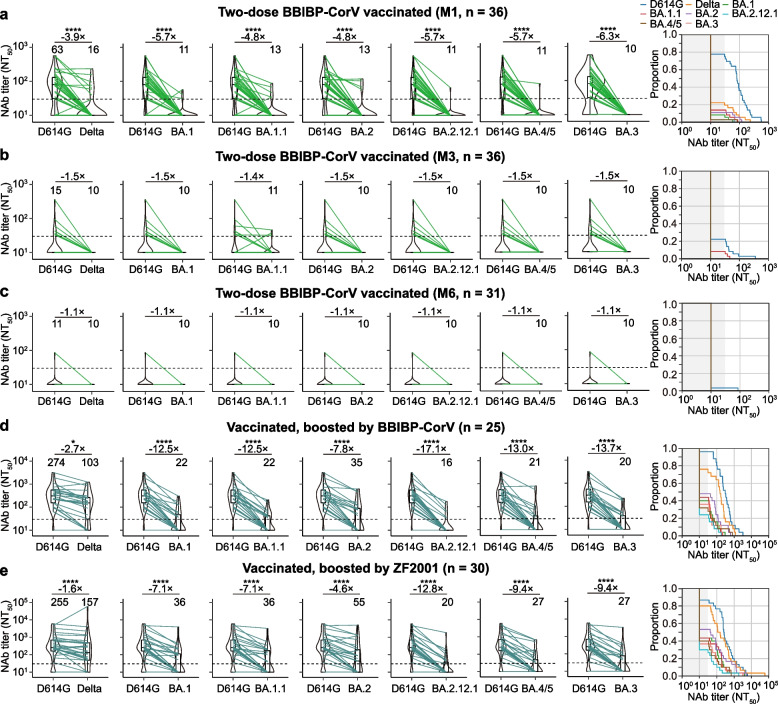
Neutralizing antibody levels in vaccinated individuals with or without booster vaccination. **a**–**c** Box-violin plots showing the median neutralizing antibody titers against the SARS-CoV-2 D614G, Delta, and Omicron subvariants in vaccinated, unboosted individuals approximately 1 month (M1) (**a**), 3 months (M3) (**b**), and 7 months (M7) (**c**) after the second dose vaccination of BBIBP-CoV (left), along with cumulative distribution function plots of titers against D614G, Delta, and Omicron subvariant (right), showing the proportion of samples at or above a given titer. **d**, **e** Box-violin plots of titers against the SARS-CoV-2 D614G, Delta, and Omicron subvariants in BBIBP-CoV booster vaccinated (**d**) and ZF2001 booster vaccinated (**e**) individuals (left), along with cumulative distribution function plots of titers against D614G, Delta, and Omicron subvariant (right), showing the proportion of samples at or above a given titer. For the box-violin plots, the median is represented by the thick black line inside the box. The geomatic mean titers (GMTs) are shown above each column. The fold change in GMT is displayed in **a**–**e**. The horizontal dotted line represents the limit of detection of 30. A two-tailed Friedman test with a false discovery rate for multiple comparisons was performed to compare Delta and Omicron subvariants to D614G in **a**–**e**. *p* values are represented as *p < 0.05, ***p < 0.001, and ****p < 0.0001. No asterisk indicates no statistical significance.

We first examined the neutralizing activity of sera collected from SARS-CoV-2-naïve individuals who were fully vaccinated with two doses of BBIBP-CorV. Serum samples were collected from 36, 36, and 31 individuals for approximately 3 weeks (median 20.5 days, interquartile range [IQR] 19.0–26.5 days, referred to M1), 3 months (median 91 days, IQR 85.5–92.8 days, referred to M3), and 7 months (median 221 days, IQR 182–249.08 days, referred to M7) after the second dose, respectively (Additional file 1: Table S1). Serum samples were tested for neutralization against D614G, Delta, and Omicron subvariants (BA.1, BA.1.1, BA.2, BA.2.12.1, BA.4/5, and BA.3). Approximately M1 after the initial two doses of BBIBP-CorV, we observed that 28 (77.8%) of 36 vaccinees had neutralizing antibody titers above the limit of detection (LOD, 30) against D614G, with a GMT of 63 (Fig. 1a). However, a substantial loss in neutralizing potency was observed against Delta and the tested Omicron subvariants (Fig. 1a). Only a few samples (8 for Delta, 3 for BA.1, 4 for BA.2, 5 for BA.1.1, and 1 for BA.2.12.1 and BA.4/5) showed titers above the LOD, and all samples showed titers below the LOD for BA.3 (Fig. 1a). For the 36 serum samples collected at M3 after the initial vaccination, only 8 (22.2%) of these samples had neutralizing antibody titers above the LOD against D614G. In contrast, the neutralizing antibody titers of all samples were below the LOD against the Delta and the tested Omicron subvariants, except for three samples that had neutralizing antibody titers above the LOD against BA.1.1 (Fig. 1b). For the 31 serum samples collected at M7 after the initial vaccination, only one of these samples had detectable pseudovirus-neutralizing antibodies against D614G. None of the serum samples had detectable neutralizing antibodies against Delta or Omicron subvariants (Fig. 1c). Collectively, these results suggest that two doses of BBIBP-CorV produced a minimal and short-term persistence of neutralizing antibody levels against VOCs.

### A third vaccination boosts the neutralizing antibody response against Delta and Omicron subvariants

Since booster vaccinations are routinely administered worldwide 6 months after full vaccination, we also measured the serum-neutralizing activity in individuals who received three homologous BBIBP-CorV or three heterologous ZF2001. After the third dose (booster) of BBIBP-CorV (*n* = 25; median 21 days, IQR 18.5–28 days after third dose vaccination), a significantly increased GMT was 274 for D614G and 103 for Delta. However, samples with neutralizing antibody titers above the LOD against Omicron subvariants showed lower activity in neutralizing Omicron subvariants, with a GMT decrease of 7.8–17.1-fold compared with D614G (Fig. 1d, left panel). The proportion of individuals with pseudovirus-neutralizing antibodies against Omicron subvariants above the LOD was ~ 40%, compared to 76.0% and 96% for Delta and D614G, respectively (Fig. 1d, right). A similar pattern was observed for the ZF2001 booster vaccination (*n* = 30; median 28 days, IQR 28–28 days after the third dose vaccination) (Fig. 1e), and there was no significant difference in GMT of 255 against D614G compared to the BBIBP-CorV booster vaccination (Additional file 1: Fig. S1). In addition, the GMT of the booster vaccination was significantly higher than that of the two doses of fully vaccinated individuals (Additional file 1: Fig. S1). Together, BBIBP-CorV or ZF2001 booster vaccination elicits an enhanced antibody response against D614G and Delta but a substantial loss in neutralizing activity against Omicron subvariants.

### Delta and BA.1 breakthrough infection increases neutralizing antibody levels against D614G and variant-specific immunity

Due to the enhanced neutralizing capacity of three separate exposures to SARS-CoV-2 by vaccination, we investigated neutralizing antibody responses and the extent of cross-neutralizing immunity in a cohort of 56 individuals with 30 Delta and 26 BA.1 breakthrough infections (Additional file 1: Table S2). Of the 30 Delta breakthrough individuals, 19 (63%) were moderate, and the other 11 (37%) were mild. Among 30 Delta breakthrough individuals, 19 (63.5%) were attributed to household transmission, two originated from each of eight households, and three originated from one household. Of the 26 BA.1 breakthrough individuals, 12 (46%) and 14 (54%) were classified as asymptomatic infection and mild-moderate disease, respectively (Additional file 1: Table S2). Among the 26 BA.1 breakthrough individuals, 17 (65.4%) were attributed to household transmission; two originated from each of five households, three originated from one household, and four originated from one household. The median age of BA.1 breakthrough infections was significantly higher than that of Delta breakthrough infections. They all had received two doses of BBIBP-CorV on average 6 months earlier. Serum samples from these individuals with breakthrough infections were collected when they were discharged from the hospital. The sampling date relative to the time of symptom onset or PCR test positivity for Delta breakthrough infection was later (median day of 41, IQR 30.0–48.0) than that for BA.1 breakthrough infection (median day of 15.5, IQR 13.0–17.0) (Additional file 1: Table S2).

**Fig. 2:**
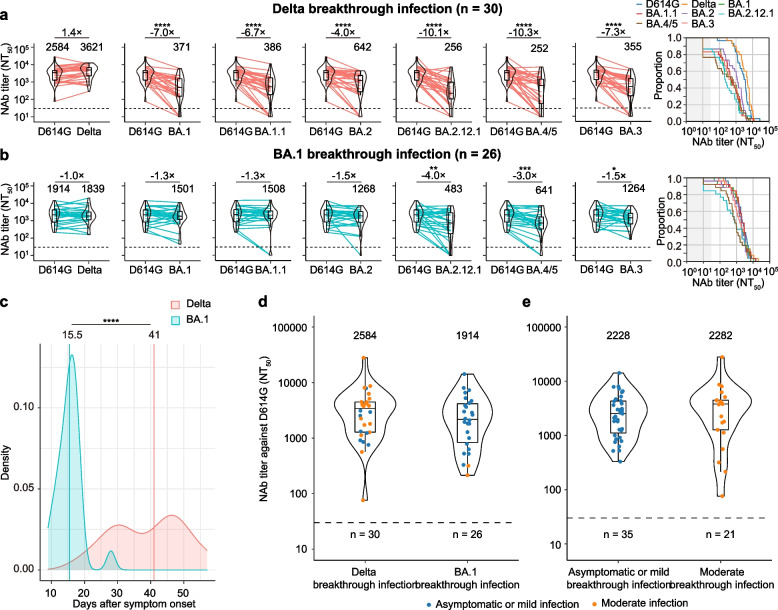
Neutralizing antibody levels in Delta and Omicron BA.1 breakthrough infections. **a** Box-violin plots of neutralizing antibody titers against Delta and Omicron subvariants compared to D614G, along with cumulative distribution function plots of titers against D614G, Delta, and Omicron subvariant (right), showing the proportion of samples at or above a given titer, in patients with Delta breakthrough infection. **b** Corresponding plots in patients with Omicron breakthrough infection using a pseudovirus neutralization assay. **c** Kernel density plot shows the distribution of collection days for samples from Delta and Omicron BA.1 breakthrough infections. All individuals with breakthrough infection in the study and available samples were collected 9–57 days after symptom onset or SARS-CoV-2 PCR test positivity when discharged from the hospital. **d** Box-violin plot showing pseudovirus-neutralizing antibody titers against D614G between Delta and Omicron BA.1 breakthrough infections. **e** Box-violin plot of pseudovirus-neutralizing antibody titers against D614G between asymptomatic or mild and moderate-severe breakthrough infections. For the box-violin plots, the median is represented by the thick black line inside the box. The geomatic mean titers (GMTs) are shown above each column for **a**, **b**, **d**, and **e**. The fold change of GMT is displayed in **a** and **b**. The horizontal dotted line represents the limit of detection of 30. A two-tailed Friedman test with a false discovery rate for multiple comparisons was performed to compare Delta and Omicron subvariants to D614G in **a** and **b**, and a two-tailed Wilcoxon rank-sum test was performed in **c**–**e**. *p* values are represented as ****p < 0.0001. No asterisk indicates no statistical significance

We first determined the breakthrough infection-neutralization antibody titer in serum samples. We observed that Delta breakthrough infections elicited 41-fold (2584 versus 63), 9.4-fold (2584 versus 274), and 10.1-fold (2584 versus 255) higher GMTs against D614G than naïve unboosted individuals and homologous BBIBP-CorV- or heterologous ZF2001-boosted individuals (Fig. 1a, d, e, left; Fig. 2a, left). Cross-neutralizing activity against Omicron subvariants was comparable (Additional file 1: Fig. S2a) but was limited as the 7.0-, 6.7-, 4.0-, 10.1-, 10.3-, and 7.3-fold reductions in BA.1 (371), BA.1.1 (386), BA.2 (642), BA.2.12.1 (256), BA.4/5 (252), and BA.3 (355) neutralization relative to D614G (2584) (Fig. 2a, left), respectively. However, neutralizing antibody titers against Omicron subvariants were significantly higher than those in naïve unboosted individuals or booster individuals (Fig. 1a–e, left panel; Fig. 2a). The proportion of serum samples with neutralizing antibodies against Omicron subvariants above the LOD of 30 was calculated at over 75% (Fig. 2a, right).

BA.1 breakthrough individuals, in contrast to Delta breakthrough individuals, exhibited relatively small increases in neutralizing titers against D614G, 30.4-fold (1914 versus 63) compared to unboosted individuals approximately 1 month after the second dose as well as 7.0-fold and 7.5-fold compared to homologous BBIBP-CorV-boosted individuals (1914 versus 274) and heterologous ZF2001-boosted individuals (1914 versus 255), respectively (Fig. 1a, d, e, left; Fig. 2b, left). Neutralizing antibody titers against Delta, BA.1, BA.1.1, and BA.2 were comparable with D614G, but neutralizing titers against BA.3 (1.5-fold), BA.2.12.1 (4.0-fold), and BA.4/5 (3.0-fold) were significantly decreased (Fig. 2b, left). BA.1 breakthrough infection resulted in 100%, ~ 92%, ~ 96%, 96%, 85%, and 96% of individuals having neutralizing antibodies against BA.1, BA.1.1, BA.2, BA.2.12.1, BA.4/5, and BA.3 above a LOD of 30 (Fig. 2b, right panel), respectively. However, the GMT of BA.4/5 was significantly lower than that of BA.1, BA.1.1, and BA.2 (Additional file 1: Fig. S2b). In addition, cross-neutralization titers against Delta were comparable to those observed for Omicron subvariants but significantly increased compared to boosting vaccinated individuals (18-fold for BBIBP-CorV and 12-fold for ZF2001).

We further compared neutralization antibody titers between Delta and BA.1 breakthrough infections, age, and disease severity. Kernel density plots showed that samples from BA.1 breakthrough infections were collected a median of 25.2 days earlier than Delta when they were discharged from the hospital, and the distribution of Delta cases was skewed toward later time points (Fig. 2c). Delta breakthrough infections resulted in equivalent neutralizing antibody titers (1.4-fold, 2584 versus 1914) against D614G but higher neutralizing antibody titers against Delta (2.0-fold, 3621 versus 1839) than BA.1 breakthrough infections, but the difference was insignificant (Fig. 2d, Additional file 1: Fig. S2c). In contrast, BA.1 breakthrough infections led to a significantly higher neutralizing antibody titer against BA.1, BA.1.1, and BA.3 subvariants (Additional file 1: Fig. S2c).

Further analysis of neutralizing antibody titers between disease severity of Delta or BA.1 infection (Fig. 2e) and age in BA.1 breakthrough individuals showed that there were no significant differences in neutralizing antibody titers between mild and moderate in Delta breakthrough individuals or asymptomatic and mild in BA.1 breakthrough individuals and between age > 18 years and < 18 years in BA.1 breakthrough individuals (Additional file 1: Fig. S3a-c), indicating that disease severity and age did not affect the antibody response in the present study. However, mild BA.1 breakthrough infection also induced a higher neutralizing antibody titer against Omicron subvariants than Delta infection (Additional file 1: Fig. S3d). Altogether, BA.1 breakthrough infections led to superior infection-neutralization capacity against Omicron subvariants and Delta.

### Potent neutralization of bebtelovimab against Delta and Omicron subvariants

**Fig. 3:**
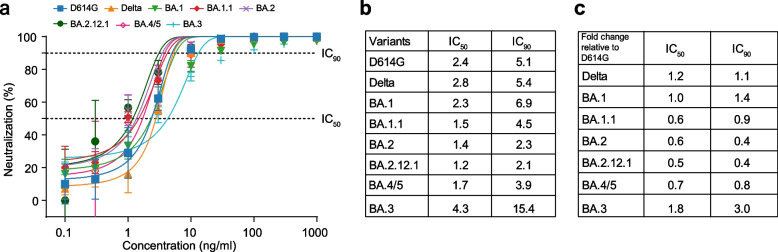
Sensitivity of Omicron subvariants to therapeutic bebtelovimab. **a** Neutralization curves against D614G, Delta, and Omicron subvariants are shown for bebtelovimab. Data are the mean ± s.d. of 2 independent experiments, each with an intra-assay duplicate. **b** Fifty percent and 90% inhibition concentration (IC_50_ and IC_90_) values against D614G, Delta, and Omicron subvariants are shown for bebtelovimab. IC_50_ and IC_90_ values were calculated from two independent experiments. **c** Fold change of IC_50_ and IC_90_ of the antibody against Delta and each Omicron subvariant compared to D614G. The horizontal dotted lines represent IC_50_ and IC_90_

Bebtelovimab (also known as LY-CoV1404), a recently authorized monoclonal therapy, showed potent neutralizing activity against BA.1, BA.1.1, and BA.2, while most mAbs have been shown to completely or partially lose neutralizing activity against BA.1 and BA.2^7,15^. Considering the decreased resistance of vaccines to BA.3, BA.2.12.1, and BA.4/5, we assessed the neutralization activity of LY-CoV1404 against BA.3, BA.2.12.1, and BA.4/5 (Fig. 3). We observed that the LY-CoV1404 monoclonal antibody effectively neutralized all tested variants, including BA.4/5 (Fig. 3a), and a similar 50% and 90% inhibition concentration (IC_50_/IC_90)_ for D614G (2.4/5.1), Delta (2.8/5.4), and BA.1 (2.3/6.9) variants, between BA.1.1 (1.5/4.5), BA.2 (1.4/2.3), BA.2.12.1 (1.2/2.1), and BA.4/5 (1.7/3.9), and the highest IC_50_/IC_90_ for the BA.3 (4.3/15.4) variant were observed (Fig. 3b). The fold changes in IC_50_/IC_90_ of LY-CoV1404 against Delta and Omicron subvariants relative to D614G showed no significant decrease in neutralizing Delta and Omicron subvariants (Fig. 3c). This finding suggests that LY-CoV1404 might be a promising therapeutic mAb against Omicron subvariants.

### Reduced fusogenicity of the S proteins of all Omicron subvariants

**Fig. 4:**
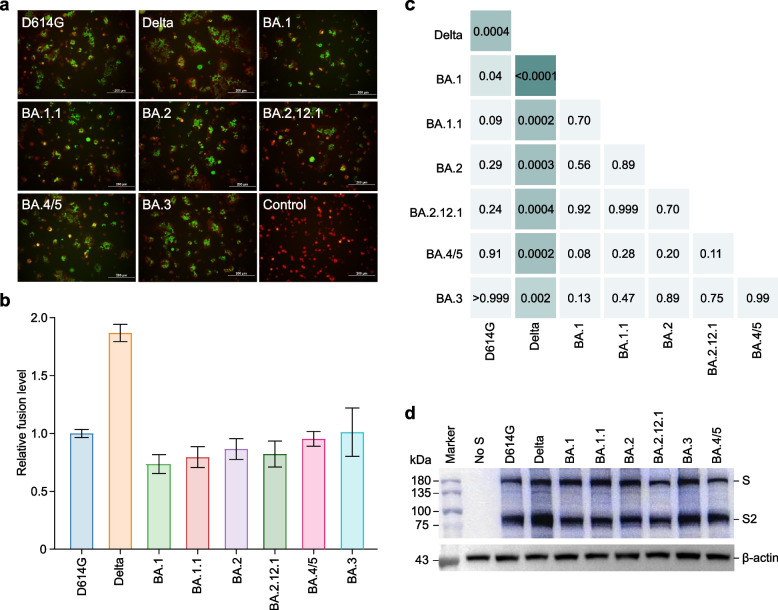
SARS-CoV-2 S-based cell fusion assay. **a** Merged images of mNeonGreen fluorescence and tdTomato red fluorescence 6 h after co-culturing. The number of nuclei (green) in a syncytium (red) represents how many cells fused to one. **b**, **c** The relative fusion levels of spike variants mediated cell fusion based on luciferase activity (**b**) and heatmap showing *p* values from pairwise comparisons of the relative fusion levels of two spike variants (**c**). **d** Representative western blots of spike variants expression. β-Actin was used as an internal control. A two-tailed Kruskal–Wallis test with a false discovery rate for multiple comparisons

The fusogenicity of SARS-CoV-2 may be associated with the degree of virus transmission, immune evasion, and pathogenicity [26–29]. We, therefore, directly assessed the fusogenicity of the S proteins of these variants by a cell-based fusion assay (Fig. 4a). Our fusion assay showed that the S proteins of all Omicron subvariants were significantly less fusogenic than Delta S (Fig. 4b, c). BA.1 S was significantly less fusogenic than the parental D614G S, and BA.1.1, BA.2, BA.2.12.1, BA.3, and BA.4/5 were comparable to D614G S (Fig. 4b, c). Next, we determined the effect of the variants and D614G on S protein cleavage by western blot assay. In line with the fusion assay, in the S-expressing cells, the level of the cleaved S2 subunit was increased for Delta S compared with the D614G-bearing parental S and S of all Omicron subvariants (Fig. 4d). Overall, these findings suggest that the S protein of all tested Omicron subvariants had comparable S cleavage and fusogenicity but was less efficient than the Delta S protein.

### Enhanced infectivity of all Omicron subvariants

**Fig. 5:**
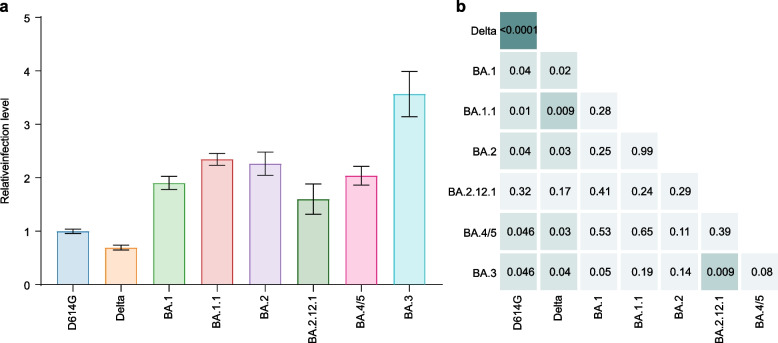
SARS-CoV-2 Omicron pseudoviruses demonstrate a substantial increase in infectivity. **a**, **b** The relative infection ability of these spike variants packaged pseudoviruses (**a**) and heatmap showing *p*-values from pairwise comparisons of the relative fusion levels between the two pseudoviruses (**b**). A two-tailed Kruskal–Wallis test with a false discovery rate for multiple comparisons

We next investigated the infectivity of Omicron subvariants compared with D614G. Remarkably, Omicron subvariant pseudovirus, except for BA.2.12.1, exhibited greater infection of target cells when compared with all other tested variants (Fig. 5a, b). We found that the Delta variant exhibited nearly 2-fold lower infection rates than D614G. Strikingly, the Omicron subvariants exhibited infection rates ~ 2- to 3.5-fold more efficient at infecting target cells than D614G and Delta. BA.1, BA.1.1, BA.2, BA.2.12.1, and BA.4/5 showed similar infection rates among the Omicron subvariants, whereas BA.3 displayed ~ 1.5-fold more efficient infection rates than the BA.1 and BA.2.12.1 subvariants. These findings strongly suggest distinct differences in infectivity according to the S sequence, with Omicron subvariants exhibiting more efficient ACE2-mediated infection than D614G and Delta.

## Discussion

In this study, we examined the neutralizing activity of SARS-CoV-2 Omicron subvariants by primary and booster vaccination and Delta and BA.1 breakthrough infections. We found that while two doses of BBIBP-CorV, achieved shortly or later after vaccination, elicited minimal neutralization of the Delta and Omicron subvariants, a booster dose, either homologous BBIBP-CorV or heterologous ZF2001, increased the neutralizing capacity against Delta and Omicron subvariants. However, an extent partial of boosting vaccinated individuals lost neutralizing activity against Omicron subvariants, which suggested that the immunogenicity of BBIBP-CorV or ZF2001 booster vaccination seems relatively low compared to mRNA boosting vaccination [30, 31]. In addition, we observed comparable antibody titers between homologous BBIBP-CorV and heterologous ZF2001 booster vaccination, which is in line with previous studies that used the same prime-boosting vaccines [22, 32] but inconsistent with previous studies that boosting heterologous vaccines (e.g., mRNA and vector-based vaccines) generates a stronger antibody response than boosting homologous vaccines [33–36]. Altogether, our results highlight the importance of vaccine boosters in enhancing immunity. However, individuals with prime BBIBP-CorV or booster vaccination with BBIBP-CorV or ZF2001 remain at risk for infection with Omicron subvariants.

We showed that the neutralizing antibody response and cross-neutralization of Omicron subvariants were substantially enhanced in Delta and BA.1 breakthrough infections [37–39]. However, in line with previous studies [37–39], sera from Delta breakthrough infections cross-neutralized all tested Omicron subvariants less well, and the titers of cross-neutralizing antibodies against Omicron subvariants were significantly lower than those against D614G. In addition, BA.2.12.1 and BA.4/5 were more resistant to humoral immunity induced by Delta breakthrough infections. Interestingly, breakthrough BA.1 infections resulted in a stronger antibody response against not only Omicron but also against a broad spectrum of D164G and Delta variants. This finding is consistent with previous studies [5, 40–42]. However, we found a modest resistance of BA.2.12.1 and BA.4/5 to the antibody response by BA.1 breakthrough infections. Antibody titers against BA.4/5 in the sera of BA.1 breakthrough infections were significantly lower than those against BA.1, BA.1.1, and BA.2. Collectively, our data suggest that Delta and BA.1 breakthrough infection provide a robust antibody response when considering the antibody levels and examining the results of the neutralizing activity to variants.

Previous studies have shown that the fusogenicity of the SARS-CoV-2 variant is closely associated with its pathogenicity and the efficiency of S1/S2 cleavage [26, 27, 29]. We observed that all Omicron subvariants showed comparable fusogenicity and S cleavage efficiency, with less fusogenic and S cleavage efficiency than Delta. However, recent studies showed that both BA.4/5 exhibited higher fusogenicity and were more pathogenic than BA.2, and both displayed higher fusogenicity and pathogenicity than BA.1 [43, 44]. In contrast, other studies have shown that the pathogenicity of BA.2 is similar to that of BA.1 [45, 46]. The different findings between our studies are likely attributed to the non-S substitutions between the genomes of the Omicron subvariants used. However, the clinical data from South Africa showed that the illnesses caused by BA.1 and BA.2 were similar [47]. Together, our data suggest that Omicron subvariants may lead to similar pathogenicity. However, because there are currently no indications that BA.4/5 subvariants are more clinically severe than previous Omicron lineages, it is necessary to re-evaluate the risk posed by subvariants BA.4 and BA.5.

Meanwhile, we observed comparable infectivity between Omicron subvariants S but increased infectivity compared to the D614G and Delta variants, which was also observed in mice and hamsters for BA.2 and BA.1 [45, 46]. Unexpectedly, the previously globally dominant Delta variant exhibited 2-fold decreased infectivity relative to the D614G and Omicron subvariants, which is inconsistent with previous studies reporting more efficient infectivity of the Delta variant that may have contributed to its rapid spread [26, 27, 48]. The high transmission rate may explain such inconsistency for the Delta variant appearing to be related to faster replication kinetics and improved replication in the nasopharynx for these viruses [49], which would not be reflected in a pseudotyped virus system or more reduction of the S1 subunit of Delta S of pseudovirus particles compared to D614G due to proteolytic cleavage, showing reduced infectivity. Together, we demonstrated a small but consistent difference between Omicron subvariants in fusogenicity and infectivity.

There are several limitations of this study. One limitation is the use of pseudotyped virus assays rather than authentic viruses for determining neutralizing antibodies, but a good correlation was established between the two assays [50]. Another limitation is that our cohort was cross-sectional based on a small sample size, which limits our ability to determine the dynamics of neutralizing antibody titers to variants across single individuals and assess the potential confounding effect on neutralizing antibody titers. In addition, the times of sample collection for Delta and BA.1 breakthrough infections were not matched, and BA.1 breakthrough samples for analysis were collected a median of 25 days earlier than Delta breakthrough samples. As a result, neutralizing antibodies to variants in BA.1 breakthrough infection did not peak antibody responses. In addition, approximately half of BA.1 breakthrough infections were less than 18 years old, although age did not affect the antibody response. Finally, this study focused on antibody responses, and vaccine- or infection-induced cellular immune responses, including memory B cells and T cells, were not assessed.

## Conclusions

We demonstrate that Omicron subvariants drastically escape vaccine-induced immunity after primary vaccination with BBIBP-CorV and exhibit decreased fusogenicity and increased pseudovirus infection in vitro. Nonetheless, receiving a third BBIBP-CorV or ZF2001 vaccine yields a relatively potent cross-neutralizing response against Delta and Omicron subvariants but is lower than Delta and BA.1 breakthrough infections. Moreover, individuals with BA.1 breakthrough infections elicited more robust neutralizing antibody titers against Omicron subvariants than Delta breakthrough, suggesting a substantial degree of cross-reactive natural immunity. These findings have important public health implications for optimizing control measures of the ongoing pandemic.

## Supplementary Information

**Additional file 1: Table S1.** Characteristics of previously uninfected individuals with unboosted and boosted vaccination; **Table S2.** Characteristics of individuals with Delta and BA.1 breakthrough infection; **Fig. S1.** Comparison of neutralizing antibody titers against D614G in primary vaccinated and boosted individuals; **Fig. S2.** Comparison of neutralizing antibody titers against the SARS-CoV-2 variants in Delta and BA.1 breakthrough infections; **Fig. S3**. Comparisons of neutralizing antibody levels in Delta and BA.1 breakthrough infections by age and disease severity of different demographics against D614G, Delta, and Omicron subvariants.

## Data Availability

The datasets supporting the conclusions of this article are included within the article and Additional file 1.

## Abbreviations

SARS-CoV-2: Severe acute respiratory syndrome coronavirus 2
VOCs: Variants of concern
S: Spike
RT–PCR: Real-time reverse transcription-polymerase chain reaction
CI: Confidence interval
IQR: Interquartile range
RBD: Receptor-binding domain
ACE2: Angiotensin-converting enzyme 2
GMT: Geometric mean titer
IC50 and IC90: Fifty percent and 90% inhibition concentration
LOD: Limit of detection

## References

[1] WHO: Tracking SARS-CoV-2 variants (August 11, 2022). 2022.

[2] Viana R, Moyo S, Amoako DG, Tegally H, Scheepers C, Althaus CL, Anyaneji UJ, Bester PA, Boni MF, Chand M (2022). Rapid epidemic expansion of the SARS-CoV-2 Omicron variant in southern Africa. Nature.

[3] Tegally H, Moir M, Everatt J, Giovanetti M, Scheepers C, Wilkinson E, et al. Emergence of SARS-CoV-2 Omicron lineages BA.4 and BA.5 in South Africa. Nat Med. 2022;28:1785-90. 10.1038/s41591-022-01911-2 PMC9499863 35760080

[4] Viana R, Moyo S, Amoako DG, Tegally H, Scheepers C, Althaus CL, et al. Rapid epidemic expansion of the SARS-CoV-2 Omicron variant in southern Africa. Nature. 2021;603:679-86. 10.1038/s41586-022-04411-y PMC8942855 35042229

[5] Wang Q, Guo Y, Iketani S, Nair MS, Li Z, Mohri H, Wang M, Yu J, Bowen AD, Chang JY (2022). Antibody evasion by SARS-CoV-2 Omicron subvariants BA.2.12.1, BA.4 and BA.5. Nature.

[6] Chen C, Nadeau S, Yared M, Voinov P, Xie N, Roemer C, Stadler T (2021). CoV-spectrum: analysis of globally shared SARS-CoV-2 data to identify and characterize new variants. Bioinformatics.

[7] Khoury DS, Cromer D, Reynaldi A, Schlub TE, Wheatley AK, Juno JA, Subbarao K, Kent SJ, Triccas JA, Davenport MP (2021). Neutralizing antibody levels are highly predictive of immune protection from symptomatic SARS-CoV-2 infection. Nat Med.

[8] Xu K, Dai L, Gao GF (2021). Humoral and cellular immunity and the safety of COVID-19 vaccines: a summary of data published by 21 May 2021. Int Immunol.

[9] Arora P, Zhang L, Krüger N, Rocha C, Sidarovich A, Schulz S, et al. SARS-CoV-2 Omicron sublineages show comparable cell entry but differential neutralization by therapeutic antibodies. Cell Host Microbe. 2022;30:1103-1111.e6. 10.1016/j.chom.2022.04.017 PMC9072809 35588741

[10] Evans JP, Zeng C, Qu P, Faraone J, Zheng YM, Carlin C, et al. Neutralization of SARS-CoV-2 Omicron sub-lineages BA.1, BA.1.1, and BA.2. Cell Host Microbe. 2022;30:1093-1102.e3. 10.1016/j.chom.2022.04.014 PMC9035359 35526534

[11] Henning G, Kanika V, Pinkus T-L, David H, Philipp S, Clara L, et al. mRNA booster immunization elicits potent neutralizing serum activity against the SARS-CoV-2 Omicron variant. Nat Med. 2022;28:477–480. 10.1038/s41591-021-01676-0 PMC8767537 35046572

[12] Hoffmann M, Krüger N, Schulz S, Cossmann A, Rocha C, Kempf A, et al. The Omicron variant is highly resistant against antibody-mediated neutralization – implications for control of the COVID-19 pandemic. Cell. 2021;185:447-456.e11. 10.1016/j.cell.2021.12.032 PMC8702401 35026151

[13] Garcia-Beltran WF, St Denis KJ, Hoelzemer A, Lam EC, Nitido AD, Sheehan ML, Berrios C, Ofoman O, Chang CC, Hauser BM (2022). mRNA-based COVID-19 vaccine boosters induce neutralizing immunity against SARS-CoV-2 Omicron variant. Cell.

[14] Planas D, Saunders N, Maes P, Guivel-Benhassine F, Planchais C, Buchrieser J, Bolland WH, Porrot F, Staropoli I, Lemoine F (2022). Considerable escape of SARS-CoV-2 Omicron to antibody neutralization. Nature.

[15] Cao Y, Wang J, Jian F, Xiao T, Song W, Yisimayi A, Huang W, Li Q, Wang P, An R (2022). Omicron escapes the majority of existing SARS-CoV-2 neutralizing antibodies. Nature.

[16] Liu L, Iketani S, Guo Y, Chan JF, Wang M, Liu L, Luo Y, Chu H, Huang Y, Nair MS (2022). Striking antibody evasion manifested by the Omicron variant of SARS-CoV-2. Nature.

[17] Carreño JM, Alshammary H, Tcheou J, Singh G, Raskin AJ, Kawabata H, Sominsky LA, Clark JJ, Adelsberg DC, Bielak DA (2022). Activity of convalescent and vaccine serum against SARS-CoV-2 Omicron. Nature.

[18] Cameroni E, Bowen JE, Rosen LE, Saliba C, Zepeda SK, Culap K, Pinto D, VanBlargan LA, De Marco A, di Iulio J (2022). Broadly neutralizing antibodies overcome SARS-CoV-2 Omicron antigenic shift. Nature.

[19] Cele S, Jackson L, Khoury DS, Khan K, Moyo-Gwete T, Tegally H, San JE, Cromer D, Scheepers C, Amoako DG (2022). Omicron extensively but incompletely escapes Pfizer BNT162b2 neutralization. Nature.

[20] Zost SJ, Gilchuk P, Case JB, Binshtein E, Chen RE, Nkolola JP, Schäfer A, Reidy JX, Trivette A, Nargi RS (2020). Potently neutralizing and protective human antibodies against SARS-CoV-2. Nature.

[21] VanBlargan LA, Errico JM, Halfmann PJ, Zost SJ, Crowe JE, Purcell LA, Kawaoka Y, Corti D, Fremont DH, Diamond MS (2022). An infectious SARS-CoV-2 B.1.1.529 Omicron virus escapes neutralization by therapeutic monoclonal antibodies. Nat Med.

[22] Zhao X, Li D, Ruan W, Chen Z, Zhang R, Zheng A, Qiao S, Zheng X, Zhao Y, Dai L (2022). Effects of a prolonged booster interval on neutralization of Omicron variant. N Engl J Med.

[23] Hacisuleyman E, Hale C, Saito Y, Blachere NE, Bergh M, Conlon EG, Schaefer-Babajew DJ, DaSilva J, Muecksch F, Gaebler C (2021). Vaccine breakthrough infections with SARS-CoV-2 variants. N Engl J Med.

[24] Juthani PV, Gupta A, Borges KA, Price CC, Lee AI, Won CH, Chun HJ (2021). Hospitalisation among vaccine breakthrough COVID-19 infections. Lancet Infect Dis.

[25] Nie J, Li Q, Wu J, Zhao C, Hao H, Liu H, Zhang L, Nie L, Qin H, Wang M (2020). Establishment and validation of a pseudovirus neutralization assay for SARS-CoV-2. Emerg Microbes Infect.

[26] Suzuki R, Yamasoba D, Kimura I, Wang L, Kishimoto M, Ito J, Morioka Y, Nao N, Nasser H, Uriu K (2022). Attenuated fusogenicity and pathogenicity of SARS-CoV-2 Omicron variant. Nature.

[27] Meng B, Abdullahi A, Ferreira I, Goonawardane N, Saito A, Kimura I, Yamasoba D, Gerber PP, Fatihi S, Rathore S (2022). Altered TMPRSS2 usage by SARS-CoV-2 Omicron impacts infectivity and fusogenicity. Nature.

[28] Zeng C, Evans JP, King T, Zheng YM, Oltz EM, Whelan SPJ, et al. SARS-CoV-2 spreads through cell-to-cell transmission. Proc Natl Acad Sci U S A. 2022;119:e2111400119. 10.1073/pnas.2111400119 PMC8740724 34937699

[29] Saito A, Irie T, Suzuki R, Maemura T, Nasser H, Uriu K, Kosugi Y, Shirakawa K, Sadamasu K, Kimura I (2022). Enhanced fusogenicity and pathogenicity of SARS-CoV-2 Delta P681R mutation. Nature.

[30] Assawakosri S, Kanokudom S, Suntronwong N, Auphimai C, Nilyanimit P, Vichaiwattana P, et al. Neutralizing activities against the Omicron variant after a heterologous booster in healthy adults receiving two doses of CoronaVac vaccination. J Infect Dis. 2022;226:1372-81. 10.1093/infdis/jiac092 35267040

[31] Costa Clemens SA, Weckx L, Clemens R, Almeida Mendes AV, Ramos Souza A, Silveira MBV, da Guarda SNF, de Nobrega MM, de Moraes Pinto MI, Gonzalez IGS (2022). Heterologous versus homologous COVID-19 booster vaccination in previous recipients of two doses of CoronaVac COVID-19 vaccine in Brazil (RHH-001): a phase 4, non-inferiority, single blind, randomised study. Lancet.

[32] Wang X, Zhao X, Song J, Wu J, Zhu Y, Li M, Cui Y, Chen Y, Yang L, Liu J (2022). Homologous or heterologous booster of inactivated vaccine reduces SARS-CoV-2 Omicron variant escape from neutralizing antibodies. Emerg Microbes Infect.

[33] Cheng SMS, Mok CKP, Leung YWY, Ng SS, Chan KCK, Ko FW, Chen C, Yiu K, Lam BHS, Lau EHY (2022). Neutralizing antibodies against the SARS-CoV-2 Omicron variant BA.1 following homologous and heterologous CoronaVac or BNT162b2 vaccination. Nat Med.

[34] Hillus D, Schwarz T, Tober-Lau P, Vanshylla K, Hastor H, Thibeault C, Jentzsch S, Helbig ET, Lippert LJ, Tscheak P (2021). Safety, reactogenicity, and immunogenicity of homologous and heterologous prime-boost immunisation with ChAdOx1 nCoV-19 and BNT162b2: a prospective cohort study. Lancet Respir Med.

[35] Atmar RL, Lyke KE, Deming ME, Jackson LA, Branche AR, El Sahly HM, Rostad CA, Martin JM, Johnston C, Rupp RE (2022). Homologous and heterologous COVID-19 booster vaccinations. N Engl J Med.

[36] Bowen JE, Addetia A, Dang HV, Stewart C, Brown JT, Sharkey WK, Sprouse KR, Walls AC, Mazzitelli IG, Logue JK (2022). Omicron spike function and neutralizing activity elicited by a comprehensive panel of vaccines. Science.

[37] Servellita V, Syed AM, Morris MK, Brazer N, Saldhi P, Garcia-Knight M, et al. Neutralizing immunity in vaccine breakthrough infections from the SARS-CoV-2 Omicron and Delta variants. Cell. 2022;185:1539-1548.e5. 10.1016/j.cell.2022.03.019 PMC8930394 35429436

[38] Wratil PR, Stern M, Priller A, Willmann A, Almanzar G, Vogel E, Feuerherd M, Cheng CC, Yazici S, Christa C (2022). Three exposures to the spike protein of SARS-CoV-2 by either infection or vaccination elicit superior neutralizing immunity to all variants of concern. Nat Med.

[39] Walls AC, Sprouse KR, Bowen JE, Joshi A, Franko N, Navarro MJ, Stewart C, Cameroni E, McCallum M, Goecker EA (2022). SARS-CoV-2 breakthrough infections elicit potent, broad, and durable neutralizing antibody responses. Cell.

[40] Khan K, Karim F, Ganga Y, Bernstein M, Jule Z, Reedoy K, Cele S, Lustig G, Amoako D, Wolter N (2022). Omicron BA.4/BA.5 escape neutralizing immunity elicited by BA.1 infection. Nat Commun.

[41] Tuekprakhon A, Nutalai R, Dijokaite-Guraliuc A, Zhou D, Ginn HM, Selvaraj M, Liu C, Mentzer AJ, Supasa P, Duyvesteyn HME (2022). Antibody escape of SARS-CoV-2 Omicron BA.4 and BA.5 from vaccine and BA.1 serum. Cell.

[42] Cao Y, Yisimayi A, Jian F, Song W, Xiao T, Wang L, Du S, Wang J, Li Q, Chen X (2022). BA.2.12.1, BA.4 and BA.5 escape antibodies elicited by Omicron infection. Nature.

[43] Yamasoba D, Kimura I, Nasser H, Morioka Y, Nao N, Ito J, Uriu K, Tsuda M, Zahradnik J, Shirakawa K (2022). Virological characteristics of the SARS-CoV-2 Omicron BA.2 spike. Cell.

[44] Kimura I, Yamasoba D, Tamura T, Nao N, Suzuki T, Oda Y, et al. Virological characteristics of the SARS-CoV-2 Omicron BA.2 subvariants including BA.4 and BA.5. Cell;185:3992-4007.e16. 10.1016/j.cell.2022.09.018 PMC9472642 36198317

[45] Uraki R, Kiso M, Iida S, Imai M, Takashita E, Kuroda M, Halfmann PJ, Loeber S, Maemura T, Yamayoshi S (2022). Characterization and antiviral susceptibility of SARS-CoV-2 Omicron BA.2. Nature.

[46] Chan JF, Hu B, Chai Y, Shuai H, Liu H, Shi J, et al. Virological features and pathogenicity of SARS-CoV-2 Omicron BA.2. Cell Rep Med. 2022;3:100743. 10.1016/j.xcrm.2022.100743 PMC9420712 36084644

[47] Wolter N, Jassat W, group D-Ga, von Gottberg A, Cohen C. Clinical severity of Omicron sub-lineage BA.2 compared to BA.1 in South Africa. Lancet. 2022;400:93-6. 10.1016/S0140-6736(22)00981-3 PMC9246473 35780802

[48] Zhang Y, Zhang T, Fang Y, Liu J, Ye Q, Ding L (2022). SARS-CoV-2 spike L452R mutation increases Omicron variant fusogenicity and infectivity as well as host glycolysis. Signal Transduct Target Ther.

[49] Planas D, Veyer D, Baidaliuk A, Staropoli I, Guivel-Benhassine F, Rajah MM, Planchais C, Porrot F, Robillard N, Puech J (2021). Reduced sensitivity of SARS-CoV-2 variant Delta to antibody neutralization. Nature.

[50] Case JB, Rothlauf PW, Chen RE, Liu Z, Zhao H, Kim AS, Bloyet L-M, Zeng Q, Tahan S, Droit L (2020). Neutralizing antibody and soluble ACE2 inhibition of a replication-competent VSV-SARS-CoV-2 and a clinical isolate of SARS-CoV-2. Cell Host Microbe.

