# Supplementary material for: Neutralization sensitivity, fusogenicity, and infectivity of Omicron subvariants
Source: Genome Med. 2022 Dec 29;14:146. doi: 10.1186/s13073-022-01151-6 (PMC9798359; doi:10.1186/s13073-022-01151-6)
Supplement: Supplementary file 1 — Additional file 1: Table S1. Characteristics of previously uninfected individuals with unboosted and boosted vaccination; Table S2. Characteristics of individuals with Delta and BA.1 breakthrough infection; Fig. S1. Comparison of neutralizing antibody titers against D614G in primary vaccinated and boosted individuals; Fig. S2. Comparison of neutralizing antibody titers against the SARS-CoV-2 variants in Delta and BA.1 breakthrough infections; Fig. S3. Comparisons of neutralizing antibody levels in Delta and BA.1 breakthrough infections by age and disease severity of different demographics against D614G, Delta, and Omicron subvariants. [file 13073_2022_1151_MOESM1_ESM.docx]

**Neutralization sensitivity, fusogenicity, and infectivity of Omicron subvariants**

Xue-Jun Wang^1†^, Lin Yao^1†^, Hong-Yun Zhang^2†^, Ka-Li Zhu^1,3†^, Jing Zhao^2†^, Bing-Dong Zhan^4^, Yi-Ke Li^1,5^, Xue-Juan He^1,5^, Cong Huang^1^, Zhuang-Ye Wang^6^, Ming-Dong Jiang^6^, Peng Yang^1^, Yang Yang^1^, Guo-Lin Wang^1^, Sheng-Qi Wang^1^, Er-Hei Dai^7^, Hui-Xia Gao^7^*, Mai-Juan Ma^1,3,5^*

^1^State Key Laboratory of Pathogen and Biosecurity, Beijing Institute of Microbiology and Epidemiology, Beijing, China

^2^Department of Respiratory and Critical Care, The Second Medical Center & National Clinical Research Center for Geriatric Diseases, Chinese PLA General Hospital, Beijing, China

^3^Department of Epidemiology and Biostatistics, School of Public Health, Anhui Medical University, Hefei, China

^4^Quzhou Center for Disease Control and Prevention, Quzhou, China

^5^School of Public Health, Zhengzhou University, Zhengzhou, China

^6^Dezhou Center for Disease Control and Prevention, Dezhou, China

^7^The Fifth Hospital of Shijiazhuang, Hebei Medical University, Shijiazhuang, China

^†^Xue-Jun Wang, Lin Yao, Hong-Yun Zhang, Ka-Li Zhu, and Jing Zhao contributed equally to this work.

*Correspondence: [swysjb@163.com](mailto:swysjb@163.com); [mjma@163.com](mailto:mjma@163.com)

**Table S1.** **Characteristics of previously uninfected individuals with unboosted and boosted vaccination.**

| Characteristics | BBIBP-CorV Vaccinated, Unboosted | | | Vaccinated, Boosted | | *p* value |
| --- | --- | --- | --- | --- | --- | --- |
|  | **M1** | **M3** | **M7** | **BBIBP-CorV** | **ZF2001** |  |
| No. of subjects | 36 | 36 | 31 | 25 | 30 |  |
| Age (median, IQR) | 44.5 (36.3-49.8) | 42.0 (31.5-52.8) | 45.0 (39.0-50.0) | 46.0 (38.5-52.0) | 39.0 (34.3-47.3) | 0.19 |
| Sex (%) |  |  |  |  |  | 0.65 |
| Male | 14 (38.9) | 17 (47.2) | 11 (35.5) | 13 (52.0) | 11 (36.7) |  |
| Female | 22 (61.1) | 19 (52.8) | 20 (64.5) | 12 (48.0) | 19 (63.3) |  |
| Interval between 2^nd^ or 3^rd^ dose and sampling (median, IQR) | 20.5 (19.0-26.5) | 91.0 (85.5-92.8) | 221 (182.0-249.0) | 21.0 (18.5-28.0) | 28.0 (28.0-28.0) |  |
| Interval between after 2^nd^ and before 3^rd^ dose (median, IQR) | NA | NA | NA | 259 (190-265.0) | 277 (237.8-298.8) | 0.003 |

IQR, interquartile range. M, month. NA, not available.

**Table S2. Characteristics of individuals with Delta and BA.1 breakthrough infection.**

| Characteristics | Delta breakthrough infection | BA.1 breakthrough infection | *p* value |
| --- | --- | --- | --- |
| No. of subjects | 30 | 26 |  |
| Age (median, IQR) | 39.0 (34.0-48.8) | 32.5 (16.0-36.8) | 0.001 |
| Age group (%) |  |  | <0.0001 |
| >18 | 30 (100) | 14 (53.8) |  |
| ≤18 | 0 | 12 (46.2) |  |
| Sex (%) |  |  | 0.08 |
| Male | 15 (50.0) | 19 (73.1) |  |
| Female | 15 (50.0) | 7 (26.9) |  |
| Disease severity (%) |  |  | <0.0001 |
| Asymptomatic | 0 | 12 (46.2) |  |
| Mild | 11 (36.7) | 12 (46.2) |  |
| Moderate | 19 (63.3) | 2 (7.6) |  |
| Interval between symptom onset or rRT-PCR positive and sampling (median, IQR) | 41.0 (30.0-48.0) | 15.5 (13.0-17.0) | <0.0001 |

IQR, interquartile range. rRT-PCR, real-time reverse transcription-polymerase chain reaction.

**
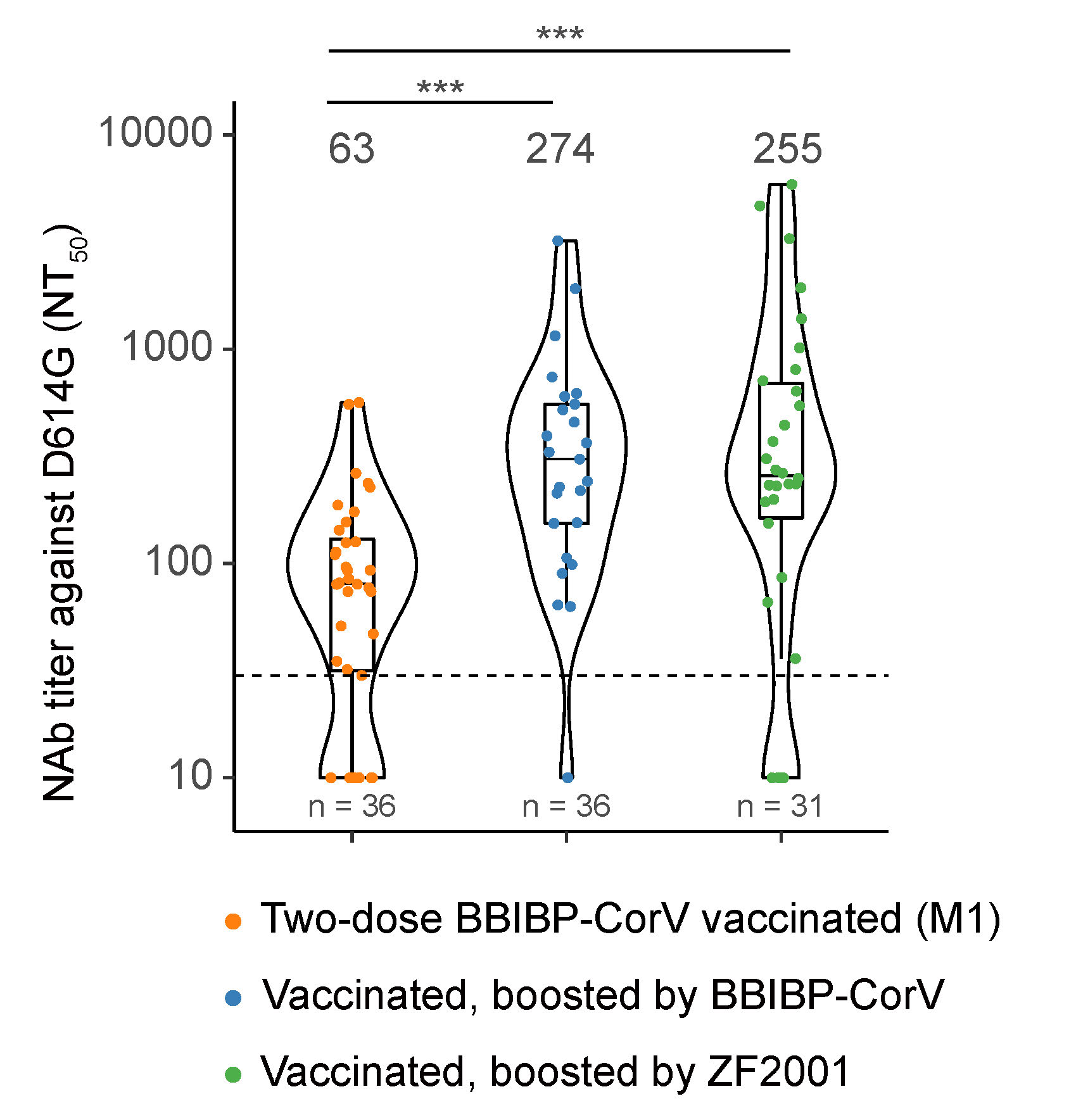
**

**Fig. S1. Comparison of neutralizing antibody titers against D614G in primary vaccinated and boosted individuals.** Box-violin plots showing median neutralizing antibody titers against D614G in two doses primary vaccinated and BBIBP-CorV or ZF2001 boosted individuals. The geomatic mean titer (GMT) are shown above each column. The horizontal dotted line represents the limit of detection of 30. A two tailed Kruskal-Wallis test was used for multiple comparisons. *p*-values are represented as ****p*<0.001.

**
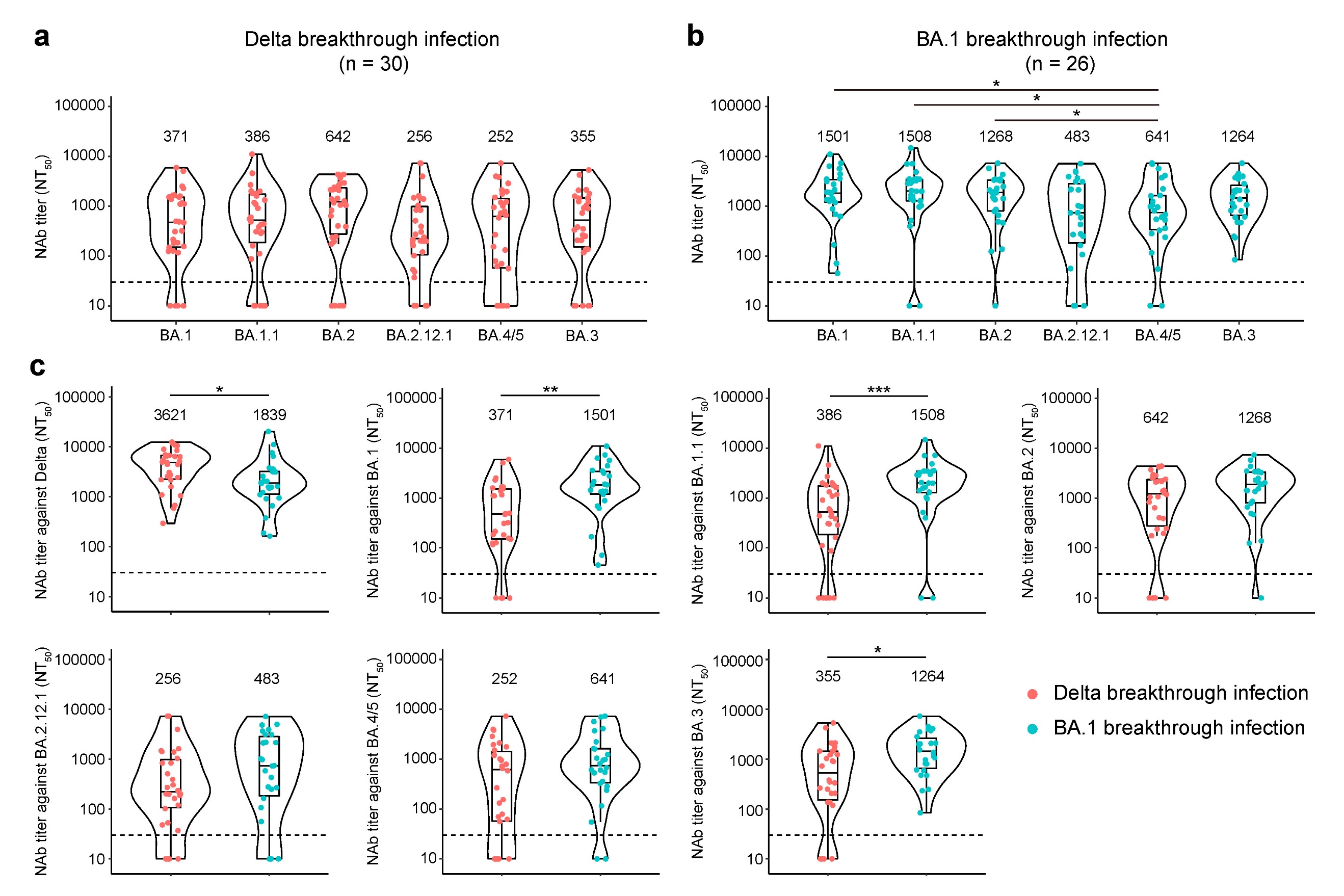
**

**Fig. S2. Comparison of neutralizing antibody titers against the SARS-CoV-2 variants in Delta and BA.1 breakthrough infections.** **a** and **b** Neutralizing antibody titers against BA.1, BA.1.1, BA.3, BA.2, BA.2.12.1 and BA.4/BA.5 in patients with Delta (**a**) and Omicron (**b**) breakthrough infections. **c** Comparisons of neutralizing antibody titers against Delta and BA.1, BA.1.1, BA.3, BA.2, BA.2.12.1, and BA.4/5 between Delta and BA.1 breakthrough infections. The geomatic mean titer (GMT) are shown above each column. The horizontal dotted line represents the limit of detection of 30. A two-tailed Friedman test with a false discovery rate was performed for multiple comparisons in **a** and **b**, and a two-tailed Wilcoxon rank-sum test was used in **c**. *p* values are represented as **p*<0.05 and ***p*<0.01.

**
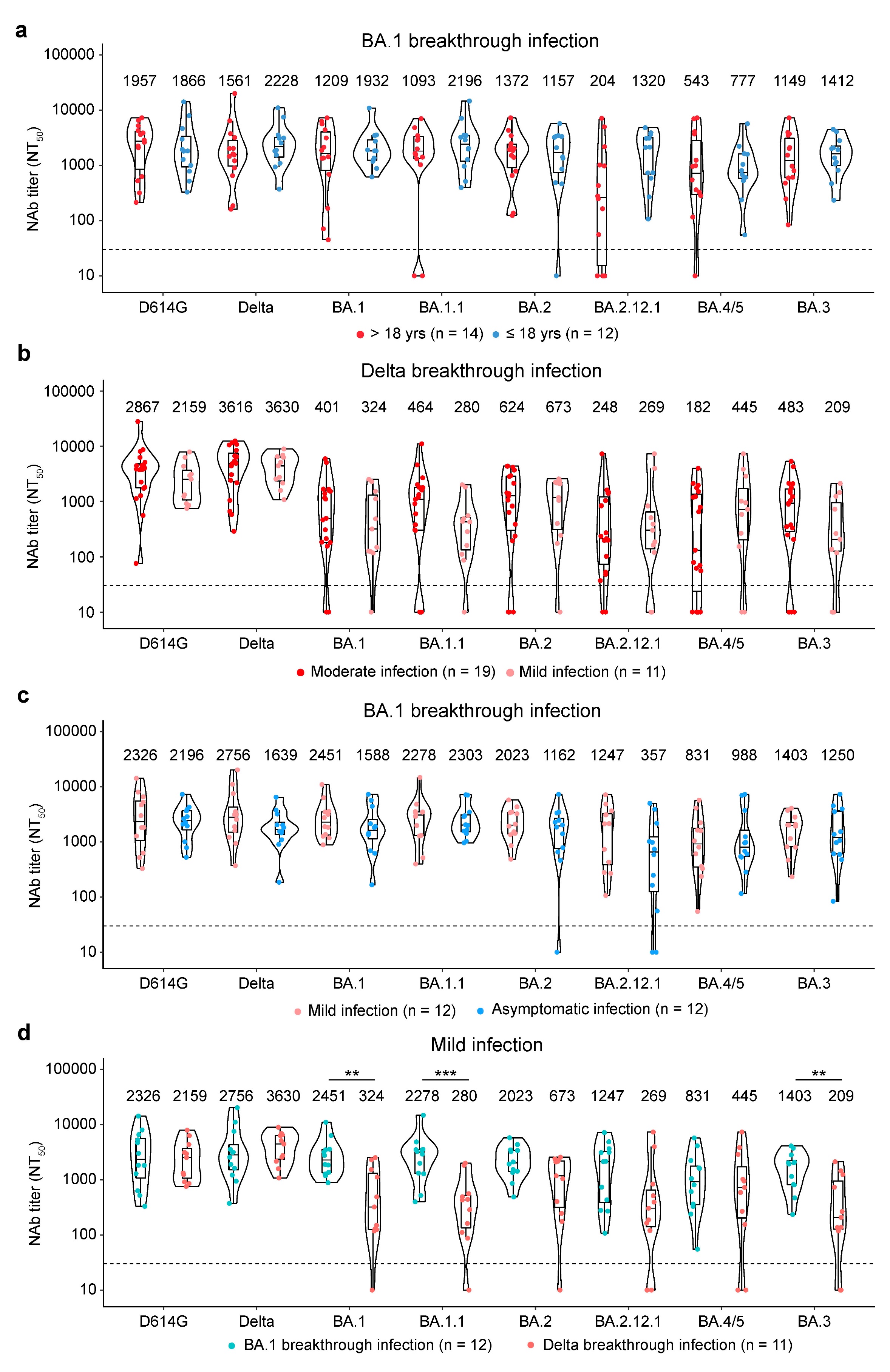
**

**Fig. S3. Comparisons of neutralizing antibody levels in Delta and BA.1 breakthrough infections by age, disease severity of different demographics against D614G, Delta, and Omicron subvariants.** **a** Comparison of the antibody titer between patients >18 years old (red, n = 14) and ≤ 18 years old (blue, n = 12) within BA.1 breakthrough infection. **b** and **c** Comparison of the antibody titers between moderate and mild patients within Delta breakthrough infection (**b**) and between mild patients and asymptomatic patients within BA.1 breakthrough infection (**c**). **d** Comparison of the antibody titers in mild patients between Delta breakthrough infection and BA.1 breakthrough infection. The horizontal dotted line represents the limit of detection of 30. The geomatic mean titer (GMT) are shown above each column. A two-tailed Wilcoxon rank-sum test was performed in **a**-**d**. *p* values are represented as ** *p*<0.01 and ****p*<0.001.
